# Bedroom environment and parental awareness surrounding junior and high school students: a parent survey study

**DOI:** 10.1007/s41105-025-00601-z

**Published:** 2025-07-22

**Authors:** Momoko Kayaba, Masahide Kondo

**Affiliations:** Department of Health Care Policy and Health Economics, Institute of Medicine, University of Tsukuba, 1-1-1 Tennodai, Tsukuba, Ibaraki 305-8577 Japan

**Keywords:** Bedroom environment, Parental awareness, Adolescents, Sleep

## Abstract

The primary objective of this study was to investigate bedroom environment and parental awareness surrounding junior and high school students. A secondary aim was to explore the relationship between these factors and sleep-related problems in adolescents. A web-based questionnaire survey was conducted in October 2023 with responses from 590 parents of junior high school students and 607 parents of high school students. The survey assessed bedroom environment, parental awareness, and adolescents’ lifestyles and sleep. Descriptive statistics were used to determine the actual conditions of the bedroom environment and parental awareness. To examine the factors associated with sleep-related problems in adolescents, we conducted logistic regression analysis. Approximately 40% of the adolescents had bedrooms with east-facing windows, and more than half used blackout curtains. Seventy percent of parents of junior high school students and 60% of parents of high school students paid attention to adolescents’ sleep duration. Logistic regression analyses showed that sleep-related problems were not significantly associated with the bedroom environment, whereas higher parental awareness of ensuring adequate sleep time was associated with reduced odds of insufficient sleep (OR = 0.36 in junior high school students, OR = 0.49 in high school students; p < 0.05) and late chronotype (OR = 0.57 and 0.63, respectively; p < 0.05) in both groups. This study clarified the bedroom environment and parental awareness of adolescents. While bedroom environment showed no association with sleep-related problems, parental awareness remained crucial in addressing these issues in both junior and high school students.

**Supplementary Information:**

The online version contains supplementary material available at 10.1007/s41105-025-00601-z.

## Introduction

Sleep-related problems associated with health and academic performance in the young population are important public health issues. In particular, the sleep–wake rhythm is delayed in the late teens because of their biological tendency to become more nocturnal [1]. Although endogenous rhythms cause them to go to bed later, the need to get up in the morning for the start of classes makes this group susceptible to insufficient sleep. Many previous studies have reported that adolescents do not get enough sleep [2], whereas 8–10 h of sleep per day is recommended for adolescents aged 14–17 y.o. [3]. A sleep survey of junior and high school students in Japan found that 28.0% of boys and 33.0% of girls slept less than six hours, and the prevalence of subjective insufficient sleep was 37.6% for boys and 38.7% for girls [4]. Insufficient sleep leads to inattentiveness, reduced neurobehavioral functioning, poor academic performance, increased risk of obesity and cardiometabolic dysfunction, sports-related injuries, mood disturbances, including increased suicidal ideation, and a higher likelihood of engaging in health-risk behaviors such as alcohol and substance use in adolescents [5, 6].

Factors contributing to insufficient sleep among adolescents include evening use of electronic media, early school start time, longer after-school activities, caffeine intake, alcohol consumption, exposure to secondhand smoke, physical and mental health issues, and biological rhythms [2, 5, 7]. Reports indicate that parental factors influence adolescent sleep; family interactions are associated with sleep duration and quality [8], lack of parental monitoring is linked to delayed bedtime [9], longer sleep duration [10], and parental expectations regarding sleep duration affect adolescents’ sleep [11]. Regarding the association between sleep environment and sleep among adolescents, higher neighborhood sound levels [12], bedtime light exposure during sleep [13], and bed/room sharing [14] were negatively associated with sleep. Although previous research has extensively examined the effects of artificial and nighttime light exposure on sleep, relatively little attention has been paid to morning sunlight exposure from natural sources. An experimental study suggested that morning bright light improves sleep quality by modulating functional connectivity in the salience network [15]. However, few studies have investigated the association between light environments—such as window orientation and curtain type—and sleep, particularly among junior and high school students. For example, if adjusting curtains can improve sleep quality by regulating morning light exposure, it would enable an environmental approach to supporting the sleep of young individuals without relying on their self-initiated efforts. This could lead to the proposal of feasible intervention strategies for populations, such as adolescents, who often struggle with behavioral change, highlighting the significance of this study as a foundation for such measures. Therefore, to clarify the actual conditions of adolescents’ bedroom environments, including aspects such as exposure to morning sunlight, this study aimed to conduct a survey targeting parents. This decision was based on the assumption that junior and high school students typically live with their parents, and that the residential environment—such as the location of the bedroom and the arrangement of furniture—is more often determined by parents than by the adolescents themselves. In addition, as the survey was conducted with parents, we examined their awareness of their adolescents’ sleep, bedroom light environment, and related factors. Furthermore, we secondarily examined the relationships between the adolescents’ sleep, their bedroom environments, and parental awareness.

## Materials and methods

### Participants and data collection

We conducted a web-based questionnaire survey using tools provided by an internet survey company with approximately 13 million registered monitors in Japan (Freeasy; iBRIDGE Company, Tokyo, JAPAN). First, we obtained responses from the first 5,000 participants by conducting screening surveys among individuals aged 35–55 years who were registered as “parents” with children. Subsequently, in October 2023, we randomly selected and distributed surveys to 900 parents of junior high school students and 900 parents of high school students, ensuring equal allocation based on the adolescents’ grade and gender, as well as the respondents’ (parents’) gender. If respondents had multiple children of the targeted age, we specified to which adolescents they should respond. The survey had a two-week response period. A total of 766 responses were obtained from parents of junior high school students and 766 from parents of high school students. Responses were considered invalid if information on adolescents’ sleep patterns and daily schedules was missing (junior high school: n = 176; high school: n = 154), or if the case was presumed to be a night high school student (i.e., with school starting in the evening or later; high school: n = 5). As a result, the number of valid responses was 590 (fathers: 50%, mothers: 50%) for parents of junior high school students, and 607 (fathers: 52%, mothers: 48%) for parents of high school students. The valid response rate was 65.6% for parents of junior high school students, and 67.4% for parents of high school students.

### Measures

We obtained data on the characteristics of respondents (age and gender) from the registration information of an internet survey company. The questionnaire was written in Japanese and consisted of the following items.

#### Bedroom environments

Parents were asked to respond to the presence of windows in the following directions: north, northeast, east, southeast, south, southwest, west, northwest, and no windows (multiple responses); types of curtains (no curtains, lace curtains, non-blackout curtains, blackout curtains, shutters, blinds, or multiple combinations); lighting colors in the bedroom (white, warm, both white and warm, or not applicable); and the number of bedroom users (single or more than two people).

#### Parental awareness

Parents were asked to respond to how much attention they paid to the following questions regarding adolescents’ sleep [1. Very attentive–5. Not attentive at all]: (a) ensuring an adequate amount of sleep, (b) maintaining a regular daily routine (avoiding staying up late), (c) lighting at night, (d)type of curtain, (e) meal times and dietary choices (caffeine intake, etc.), (f) use of smartphones or games before bedtime, and (g) going out at night. The level of parental awareness was converted into high attention for “1. Very attentive,” and “2. Somewhat attentive”, and low attention for “3. Neutral” to “5. Not attentive at all” to compare groups and examine their association with sleep-related problems.

#### Social schedule, Sleep patterns, and sleep-related problems

Bedtime and wake-up time on weekdays and weekends, morning departure time, and class start time were reported by parents. Morning departure time was selected as a key variable representing social demands, as it reflects not only school start times but also students’ individual schedules, including early extracurricular activities. Since commuting times and school schedules can vary considerably, departure time was considered to have a more direct influence on wake-up time than commuting duration itself.

Sleep duration and the midpoint of sleep were calculated using bedtime and wake-up time. For sleep-related problems, extended sleep duration of more than two hours on weekends compared to on weekdays was defined as “insufficient sleep”; the midpoint of sleep on weekdays was later than median was defined as “late chronotype”; responses regarding the frequency of skipping breakfast (always/sometimes/not at all) include “always” and “sometimes.” was defined as “skipping breakfast.”

### Statistical analysis

Descriptive statistics were used to determine the actual conditions of the bedroom environment, parental awareness, and sleep patterns. Representative values are shown as the mean and standard deviation. The chi-square test and Mann–Whitney U test were performed to compare groups. Spearman’s rank correlation coefficient and a logistic regression model were used to identify the factors associated with sleep among adolescents. In the logistic regression model, the degree of association is represented as the adjusted odds ratio (aOR) and 95% confidence interval (95%CI). The Hosmer–Lemeshow test was conducted to assess the fit of the model. Statistical significance was set at P < 0.05. All analyses were conducted separately for junior high school and senior high school students as subgroup analyses, in order to account for developmental and lifestyle differences. Statistical analyses were performed using IBM SPSS Statistics Version 25.0 (IBM Corporation, Armonk, NY, USA).

### Ethics

The Ethical Committee of the University of Tsukuba granted ethical approval for this study (No. 1912). Informed consent was obtained from all the participants.

## Results

### Characteristics of adolescents reported by parents

Table 1 shows the distribution of the target junior high school students (16% 1st-year boys, 18% 2nd-year boys, 17% 3rd-year boys, 16% 1st-year girls, 18% 2nd-year girls, and 16% 3rd-year girls) and high school students (17% 1st-year boys, 17% 2nd-year boys, 15% 3rd-year boys, 16% 1st-year girls, 17% 2nd-year girls, and 18% 3rd-year girls).

**Table 1:** Characteristics of adolescents reported by parents

Junior high school students (n = 590)	
1st-year boys (%)	16
2st-year boys (%)	18
3st-year boys (%)	17
1st-year girls (%)	16
2st-year girls (%)	18
3st-year girls (%)	16
High school students (n = 607)	
1st-year boys (%)	17
2nd-year boys (%)	17
3rd-year boys (%)	15
1st-year girls (%)	16
2nd-year girls (%)	17
3rd-year girls (%)	18

Generally, junior high school students were aged 1st-year (12–13y.o), 2nd-year (13–14y.o), and 3rd-year (14–15y.o), and high school students were aged 1st-year (15–16y.o), 2nd-year (16–17y.o), and 3rd-year (17–18y.o) in Japan

### Parent-reported adolescents’ bedroom environment, social schedule, sleep patterns, and sleep-related problems

Table 2 shows the bedroom environment, social schedule, sleep patterns, and sleep-related problems of the junior and high school students. Details of the bedroom environment (direction of windows, type of curtain, and lighting color) are also provided in Supplemental Table S1.

**Table 2:** Bedroom environment, social schedule, sleep patterns, and sleep-related problems among junior high and high school students

		Junior high school students (n = 590)	High school students (n = 607)	*p-* value	Effect size	
Bedroom environment						
Direction of the windows						
Non-east-facing	(%)	60.8	60.0	0.76	< 0.01	
East-facing	(%)	39.2	40.0			
Type of curtain						
Non-blackout curtain	(%)	43.2	43.8	0.94	< 0.01	
Blackout curtain	(%)	56.8	56.2			
Lighting color						
White	(%)	54.9	53.2	0.76	< 0.01	
Warm	(%)	25.8	26.0			
Both or others	(%)	19.3	20.8			
Number of bedroom users						
Single	(%)	66.6	76.3	< 0.01	0.11	*
More than two people	(%)	33.4	23.7			
Social schedule						
Morning departure time	(h:m)	07:40h (05:40–09:00h)	07:34h (06:00–09:45h)	< 0.01	0.01	
Class start time	(h:m)	08:36h (08:00–09:00h)	08:42 (08:00–10:00h)	< 0.01	0.16	*
Sleep patterns						
Bedtime on weekdays	(h:m)	22:52h (20:00–02:00h)	23:27h (20:00–03:00h)	< 0.01	0.30	**
Wake-up time on weekdays	(h:m)	06:39 h (05:00–08:30h)	06:32 h (03:00–12:00h)	< 0.01	0.01	
Sleep duration on weekdays	(h)	7.8 (0.9)	7.1 (1.1)	< 0.01	0.34	**
Bedtime on weekends	(h:m)	23:13 (20:00–4:00)	23:52 (20:30–4:00)	< 0.01	0.28	*
Wake-up time on weekends	(h:m)	08:06h (04:00–15:00h)	08:29 h (03:00–16:00h)	< 0.01	0.13	*
Sleep duration on weekends	(h)	8.9 (1.3)	8.6 (1.5)	< 0.01	0.10	*
Extension of sleep duration on weekends	(h)	1.1 (1.3)	1.5 (1.5)	< 0.01	0.16	*
Midpoint of sleep on weekends	(h:m)	03:40h (01:00–08:00h)	04:11h (01:00–10:00h)	< 0.01	0.22	*
Sleep-related problems						
Insufficient sleep	(%)	24.1	40.4	< 0.01	0.17	*
Late chronotype	(%)	60.8	60.6	–	–	
Skipping breakfast	(%)	22.2	31.1	< 0.01	0.10	*

Representative values are presented as the mean and standard deviation, and times are presented as the mean and range

Effect sizes are reported as absolute values. For chi-square tests, Phi or Cramér’s V was used; for tests involving Z values, effect size r was calculated as r = Z/√n

^*^Indicates a small effect size (r ≥ 0.1), and ** indicates a medium effect size (r ≥ 0.3)

Late chronotype: No statistical test was performed because the data were dichotomized at the median

The details of the bedroom environment (direction of windows, type of curtain, and lighting color) are provided in the supplemental table S1

Bedroom environments did not differ between junior and high school students, except for the number of bedroom users, where students who used bedrooms alone were more prevalent among high school students. The class start time was later and the morning departure time was earlier among high school students than among junior high school students. Sleep patterns differed between junior and high school students: high school students went to bed later and woke up earlier on weekdays, slept for shorter hours on weekdays, and slept longer on weekends. Additionally, insufficient sleep and skipping breakfast were more prevalent among the high school students.

### Parental awareness

**Fig. 1:**
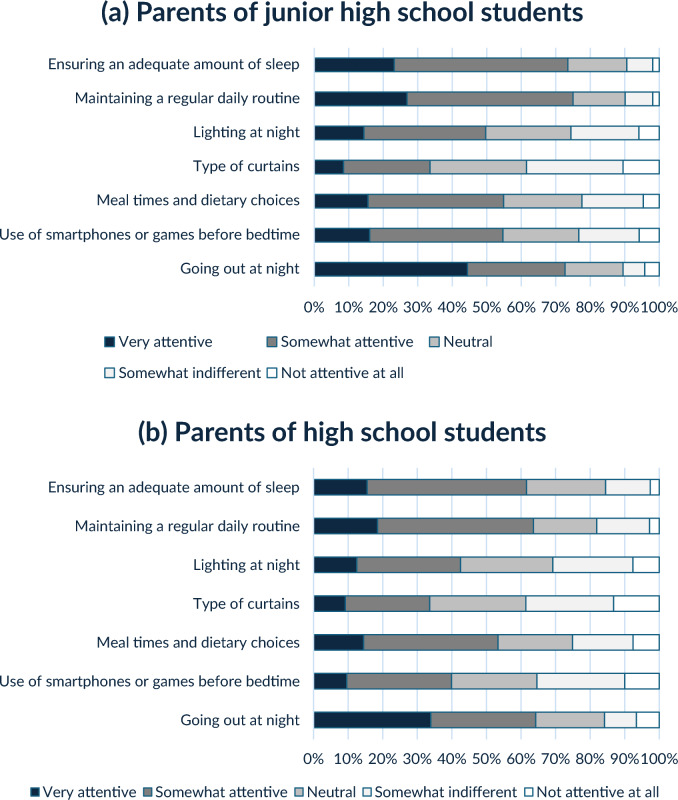
Parental awareness

Figure 1 shows parental awareness of each question. Parental awareness varied between junior and high school students; the percentage of high attention was significantly lower for high school students than for junior high school students in the items of “ensuring an adequate amount of sleep (62% VS 74%),” “maintaining a regular daily routine (64% VS 75%),” “lighting at night (43% VS 50%),” “use of smartphones or games before bedtime (40% VS 44%),” and “going out at night (64% VS 73%).”

### Factors associated with adolescents’ sleep

#### Correlation between social schedule and sleep patterns

The correlation coefficients between morning departure time, class start time, and sleep patterns are shown in Table 3. Morning departure time was significantly correlated with wake-up time (junior high school students: r_s_ = 0.58, p < 0.001; high school students: r_s_ = 0.60, p < 0.001) and sleep duration on weekdays (r_s_ = 0.12, p < 0.001; r_s_ = 0.32, p < 0.001) in both the groups. Among junior high school students, a significant positive correlation was found between morning departure time and weekday bedtime (r_s_ = 0.20, p < 0.001); however, no such correlation was observed among high school students.

**Table 3:** The Spearman’s correlation coefficients between social schedule and sleep patterns

	Junior high school students (n = 590)		High school students (n = 607)	
	Morning departure time	Class start time	Morning departure time	Class start time
Class start time	0.31^**^	1.00	0.33^**^	1.00
[Weekdays]				
Bedtime	0.20^**^	0.07	0.04	0.08^*^
Wake-up time	0.58^**^	0.14^**^	0.60^**^	0.18^**^
Sleep duration	0.12^**^	0.02	0.32^**^	0.05
[Weekends]				
Extension of sleep duration	−0.05	−0.05	−0.14^**^	−0.08

^**^ p < 0.01, * p < 0.05

#### Factors associated with sleep-related problems

*(1) Insufficient sleep:* Insufficient sleep was associated with parental attention to ensure an adequate amount of sleep in junior high school students (aOR = 0.36, 95% CI 0.22–0.61, p < 0.001) and high school students (aOR = 0.49, 95% CI 0.32–0.77, p = 0.002), and morning departure time (aOR = 0.63, 95% CI 0.43–0.93, p = 0.020) among high school students, while it was not associated with bedroom environment (Table 4).

**Table 4:** Logistic regression model for associated factors with insufficient sleep

Explanatory variables	Junior high school students (n = 590)		High school students (n = 607)	
	aOR	95%CI	aOR	95%CI
Bedroom environment				
Direction of the window				
Non-east-facing	1.00		1.00	
East-facing	0.84	0.50–1.39	1.21	0.78–1.90
Type of curtain				
Non-blackout curtain	1.00		1.00	
Blackout curtain	1.33	0.80–2.21	1.05	0.68–1.62
Lighting color				
White	1.00		1.00	
Warm	0.80	0.47–1.38	1.09	0.69–1.72
Number of bedroom users				
Single	1.00		1.00	
More than two people	0.80	0.50–1.38	1.41	0.83–2.39
Social schedule				
Morning departure time	0.62	0.33–1.17	0.63^**^	0.43–0.93
Parental awareness				
Ensure an adequate amount of sleep				
Low attention	1.00		1.00	
High attention	0.36^***^	0.22–0.61	0.49^**^	0.32–0.77

^***^ p < 0.001, ** p < 0.05

aOR indicated OR adjusted for all other explanatory variables in addition to gender-grade group

Hosmer–Lemeshow test: p > 0.05

*(2) Late chronotype:* Late chronotype was associated with parental attention to ensure an adequate amount of sleep (junior high school students, aOR = 0.57, 95% CI 0.34–0.97, p = 0.038; high school students, aOR = 0.63, 95% CI 0.41–0.99, p = 0.043) and morning departure time (junior high school students, aOR = 2.10, 95% CI 1.17–3.75, p = 0.013; high school students, aOR = 1.52, 95% CI 1.04–2.22, p = 0.030) in both groups of students, while it was not associated with bedroom environment (Table 5).

**Table 5:** Logistic regression model for associated factors with late chronotype

Explanatory variables	Junior high school students (n = 590)		High school students (n = 607)	
	aOR	95%CI	aOR	95%CI
Bedroom environment				
Direction of the window				
Non-east-facing	1.00		1.00	
East-facing	1.35	0.85–2.13	0.99	0.65–1.51
Type of curtain				
Non-blackout curtain	1.00		1.00	
Blackout curtain	0.72	0.46–1.13	0.97	0.64–1.48
Lighting color				
White	1.00		1.00	
Warm	1.19	0.73–1.92	0.73	0.47–1.13
Number of bedroom users				
Single	1.00		1.00	
More than two people	0.76	0.47–1.23	0.86	0.51–1.45
Social schedule				
Morning departure time	2.10^**^	1.17–3.75	1.52^**^	1.04–2.22
Parental awareness				
Ensure an adequate amount of sleep				
Low attention	1.00		1.00	
High attention	0.57^**^	0.34–0.97	0.63^**^	0.41–0.99

^***^ p < 0.001, ** p < 0.05

aOR indicated OR adjusted for all other explanatory variables in addition to gender-grade group

Hosmer–Lemeshow test: p > 0.05

*(3) Skipping breakfast:* Skipping breakfast was associated with parental attention to ensure an adequate amount of sleep (aOR = 0.55, 95% CI 0.33–0.93, p = 0.024) and lighting color (warm color) (aOR = 1.71, 95% CI 1.04–2.84, p = 0.036) only among junior high school students (Table 6). No association with skipping breakfast was observed among high school students.

**Table 6:** Logistic regression model for associated factors with skipping breakfast

Explanatory variables	Junior high school students (n = 590)		High school students (n = 607)	
	aOR	95%CI	aOR	95%CI
Bedroom environment				
Direction of the window				
Non-east-facing	1.00		1.00	
East-facing	0.95	0.58–1.55	1.28	0.80–2.03
Type of curtain				
Non-blackout curtain	1.00		1.00	
Blackout curtain	0.66	0.41–1.29	0.98	0.62–1.55
Lighting color				
White	1.00		1.00	
Warm	1.72^**^	1.04–2.84	1.01	0.63–1.64
Number of bedroom users				
Single	1.00		1.00	
More than two people	0.76	0.44–1.29	1.00	0.57–1.77
Social schedule				
Morning departure time	1.11	0.59–2.06	1.18	0.78–1.77
Parental awareness				
Ensure an adequate amount of sleep				
Low attention	1.00		1.00	
High attention	0.55^**^	0.33–0.93	0.97	0.61–1.57

^***^ p < 0.001, ** p < 0.05

aOR indicated OR adjusted for all other explanatory variables in addition to gender-grade group

Hosmer–Lemeshow test: p > 0.05

## Discussion

This study investigated the bedroom environment and parental awareness of adolescents and its association with adolescents’ sleep. We hypothesized that improving the bedroom environment would help resolve sleep-related problems among adolescents. However, contrary to our expectations, no significant association was found between the bedroom environment and sleep. More notably, our results indicate that social schedule and parental awareness have a greater influence on adolescent sleep than bedroom environment. One key finding was the positive correlation between wake-up time on weekdays and morning departure time for both junior and high school students. Among junior high school students, earlier departure time was associated with earlier bedtime on weekdays. However, for high school students, despite an earlier morning departure time, bedtime did not change. Furthermore, the correlation between morning departure time and sleep duration was stronger for high school students (r_s_ = 0.32) than for junior high school students (r_s_ = 0.12). High school students compensated for insufficient sleep on weekdays by extending their weekend sleep duration. Additionally, insufficient sleep and skipping breakfast were more prevalent among the high school students. For junior high school students, an earlier morning departure time may be manageable by going to bed earlier, suggesting a potential for self-directed solutions. However, this may not be feasible for high school students, who often face longer commutes due to reliance on trains or buses, resulting in earlier departure time compared to junior high school students. Our study also revealed that high school students departed significantly earlier, although both junior and high school students left for school around 07:30 h–07:40 h. The wake-up time for both groups was approximately 06:30 h. According to the recommended sleep duration [2], adolescents in this age group need 8–10 h of sleep. To meet this recommendation, students would need to go to bed between 20:30 h and 22:30 h if they awoke at 06:30 h. However, the results of this study showed that high school students went to bed around 23:30 h, making it unrealistic to encourage bedtime between 20:30 h and 22:30 h. This discrepancy is partly due to the biological tendency of adolescents, particularly those in their late teens, to shift toward a later chronotype, making it difficult for them to fall asleep earlier [1]. Therefore, aligning school schedules with biological rhythms is crucial. Previous studies have shown that delayed high school start times improve sleep, reduce daytime sleepiness [16, 17] and enhance academic performance [18]. Later start times have also been linked to lower migraine frequency [19]. In Hong Kong, a 1-h delay in school start time was associated with improved sleep duration, mental health, and life satisfaction, as well as increased breakfast consumption and decreased unexcused tardiness, absences, and clinic visits [20]. However, another study suggested that, although later start times may delay sleep onset, they do not necessarily extend sleep duration, suggesting that delayed start times may be a necessary but insufficient measure to ensure adequate sleep [21]. Although society expects students to have sufficient sleep time, simply providing sleep time may not resolve the issue of insufficient sleep. In Japan, where sleep duration is the shortest internationally [22], it is necessary to establish an understanding of and practice to ensure adequate sleep duration, both socially and individually. Furthermore, longer commuting time has been linked to depression among high school students in Japan [23], highlighting the need for measures that allow sufficient sleep and support for overall health. It is essential to raise awareness regarding the importance of mental health and sleep hygiene among schools, parents, and students. Schools should consider students’ biological rhythms when planning school schedules, while personal strategies that students and parents can adopt include ensuring that the school’s schedule allows for the maintenance of a healthy lifestyle, including sufficient sleep duration, when selecting schools.

The study found that insufficient sleep, late chronotype, and skipping breakfast were associated with parental attention to ensure an adequate amount of sleep. A previous review of parental factors influencing adolescent sleep suggested that bedtime rules and restrictions on the nighttime use of electronic devices are linked to sleep duration in adolescents [24]. This study further suggests that parents may play a key role in shaping their adolescents’ sleep habits, as there is a notable similarity in sleep patterns between parents and their adolescents, along with the understanding that adolescent behavioral patterns develop based on social learning theory. Therefore, promoting healthy sleep habits among parents is crucial for interventions aimed at addressing sleep-related problems among adolescents. A study conducted in Dutch high schools found that parental subjective norms and perceived behavioral control were associated with longer sleep duration in adolescents [25]. These findings suggest that sleep education for both students and their parents could be an effective intervention to promote adequate sleep durations. Figure 1 shows that parental awareness of sleep issues was weaker among high school students than junior high school students, which aligns with the increasing independence of adolescents in this age group. However, it is noteworthy that parental concern about sleep-related.

problems still plays a role among high school students, underscoring the continued importance of parental involvement even as adolescents strive for greater autonomy. Ensuring adequate sleep, maintaining a healthy chronotype, emphasizing the importance of breakfast, and fostering healthy lifestyle habits at a young age are crucial for long-term health and wellness. Therefore, these factors are an important public health concern.

Contrary to our hypothesis, the association between the bedroom environment (e.g., window direction, curtain type, and lighting color) and sleep was not confirmed. Instead, this study found that social schedule and parental awareness had a more significant impact on sleep. Although an association was observed between lighting color and skipping breakfast among junior high school students, the reasons for this relationship remain unclear. Future research should incorporate objective measures such as light intensity (lux) and other environmental factors.

This study has several limitations. First, as the survey was conducted by an online research company, the generalizability of the findings may be limited. Second, although we assessed the sleep pattern of junior and high school students based on parent-reported data, which may not fully reflect the students’ actual sleep behaviors, a previous study have shown that parent reports tend to overestimate sleep duration compared to objective measures such as actigraphy [26]. Considering this, it is possible that the prevalence of insufficient sleep among adolescents may be underestimated. Nevertheless, the differences in sleep patterns and sleep-related problems observed between junior and high school students were consistent with well-established findings, suggesting that the results are reasonable and reliable. Furthermore, our study provides valuable insights into bedroom environments and parental awareness, contributing important evidence to this field despite inherent limitations. Third, a comprehensive understanding of adolescents’ sleep would require direct information on their daily life, school activities, and time use. Therefore, future research is needed to conduct bedroom environment surveys targeting both adolescents and their parents, taking into account overall lifestyle and physical and mental health.

## Conclusion

This study highlights the greater influence of social schedule and parental awareness on adolescent sleep-related problems than bedroom environment. As the study revealed that high school students, in particular, face challenges with sleep duration due to their biological clocks and social commitments, interventions addressing sleep issues should target both the social (e.g., school schedules) and individual (e.g., parental involvement, education for students and parents, school selection). In addition, parental awareness remains crucial in addressing sleep-related problems in both age groups. Future research should explore additional environmental factors and employ objective measures to gain deeper insights into how these factors influence adolescent sleep.

## Supplementary Information

Below is the link to the electronic supplementary material.

Supplementary file1 (DOCX 13 KB)
