# Supplementary material for: Bedroom environment and parental awareness surrounding junior and high school students: a parent survey study
Source: Sleep Biol Rhythms. 2025 Jul 22;24(1):39–48. doi: 10.1007/s41105-025-00601-z (PMC12804560; doi:10.1007/s41105-025-00601-z)
Supplement: Supplementary file 1 — Supplementary file1 (DOCX 13 KB) [file 41105_2025_601_MOESM1_ESM.docx]

Supplemental Table S1. Details of the bedroom environment among junior and high school students

|  |  | Junior high school students  (n=590) | High school  students  (n=607) |
| --- | --- | --- | --- |
| No window in the bedroom  (Exclusive choice) | (%) | 2.5 | 2.1 |
| Window orientation  (Multiple choice) |  |  |  |
| North | (%) | 19.8 | 23.2 |
| Northeast | (%) | 7.3 | 9.2 |
| East | (%) | 22.7 | 23.7 |
| Southeast | (%) | 10.0 | 9.1 |
| South | (%) | 38.6 | 35.6 |
| Southwest | (%) | 8.1 | 6.1 |
| West | (%) | 18.3 | 17.1 |
| Northwest | (%) | 3.2 | 3.8 |
| Types of curtains |  |  |  |
| No curtains | (%) | 6.3 | 5.1 |
| Only lace curtains | (%) | 3.7 | 5.6 |
| Only non-blackout curtains | (%) | 30.2 | 30.0 |
| Only blackout curtains | (%) | 44.6 | 45.6 |
| Only shutters or blinds | (%) | 2.2 | 3.0 |
| Others | (%) | 1.5 | 1.3 |
| Multiple combinations | (%) | 11.5 | 9.4 |
| Lighting colors |  |  |  |
| Only white | (%) | 54.9 | 53.2 |
| Only warm | (%) | 25.8 | 26.0 |
| Both white and warm | (%) | 15.9 | 17.5 |
| Not applicable | (%) | 3.4 | 3.3 |
